# DNA barcoding for species delimitation of the freshwater leech genus *Glossiphonia* from the Western Balkan (Hirudinea, Glossiphoniidae)

**DOI:** 10.3897/BDJ.9.e66347

**Published:** 2021-09-15

**Authors:** Milica Jovanović, Elisabeth Haring, Helmut Sattmann, Clemens Grosser, Vladimir Pesic

**Affiliations:** 1 Department of Biology, Faculty of Natural Science and Mathematics, University of Montenegro, Džordža Vašingtona bb, 81000, Podgorica, Montenegro Department of Biology, Faculty of Natural Science and Mathematics, University of Montenegro, Džordža Vašingtona bb, 81000 Podgorica Montenegro; 2 Department of Evolutionary Biology, University of Vienna, Althanstraße 14, 1090, Vienna, Austria Department of Evolutionary Biology, University of Vienna, Althanstraße 14, 1090 Vienna Austria; 3 Central Research Laboratories, Natural History Museum Vienna, Burgring 7, 1010, Vienna, Austria Central Research Laboratories, Natural History Museum Vienna, Burgring 7, 1010 Vienna Austria; 4 3rd Zoological Department, Natural History Museum Vienna, Burgring 7, Vienna, Austria 3rd Zoological Department, Natural History Museum Vienna, Burgring 7 Vienna Austria; 5 4 Bernd-Blindow-Schule Leipzig, Comeniusstraße 17, 04315, Leipzig, Germany 4 Bernd-Blindow-Schule Leipzig, Comeniusstraße 17, 04315 Leipzig Germany

**Keywords:** DNA barcoding, COI, freshwater leeches, Glossiphoniidae, phylogeny, species delimitation

## Abstract

Glossiphoniid leeches are a diverse group and sometimes abundant elements of the aquatic fauna inhabiting various types of freshwater habitats. In this study, we sampled leeches of the genus *Glossiphonia* from the Western Balkan in order to test the suitability of the mitochondrial cytochrome c oxidase subunit 1 (COI) marker sequence for species delimitation. Morphological analysis revealed the presence of four taxa, *G.complanata* with two subspecies, *G.c.complanata* and *G.c.maculosa*, the latter an endemic of Ohrid Lake, *G.nebulosa* and endemic *G.balcanica*. In total, 29 new barcodes of *Glossiphonia* were sequenced in the course of this study and compared with the available molecular dataset of the latter genus from GenBank/BOLD databases. The applied ASAP distance-based species delimitation method for the analysed dataset revealed an interspecific threshold between 4-8% K2P distance as suitable for species identification purposes of the Western Balkan *Glossiphonia* species. Our study revealed that morphologically identified taxa as *G.nebulosa* and *G.concolor* each consists of more than one clearly different phylogenetic clade. This study contributes to a better knowledge of the taxonomy of glossiphoniid leeches and emphasises future work on the revision of this genus using a standard molecular COI marker in species identification.

## Introduction

Species of the family Glossiphoniidae Vaillant, 1890 are generally small, dorsoventrally flattened leeches, distributed in freshwater ecosystems on all continents except Antarctica (Nesemann and Neubert 1999, Kaygorodova et al. 2020). Representatives of these leeches are normally found feeding on the blood of turtles or amphibians and, as vectors of apicomplexan blood parasites, they play an important role in aquatic ecosystems (Nesemann and Neubert 1999, Siddall et al. 2005, Chiangkul et al. 2021). Some species of the genera *Helobdella* Blanchard, 1896 and *Glossiphonia* Johnson, 1816 feed on the haemolymph of aquatic oligochaetes and snails (Siddall et al. 2005).

Distribution and species boundaries of the leeches of the genus *Glossiphonia*, the most diverse genus of the family, have been studied by several authors by means of the DNA barcode region of the mitochondrial cytochrome c oxidase subunit 1 gene (COI) as a genetic marker (e.g. Siddall et al. 2005, Oceguera-Figueroa and León-Règagnon 2014, Pérez-Flores et al. 2016, Mack and Kvist 2019, Kaygorodova et al. 2020). An integrative approach that combines morphological examination and molecular genetic data had helped to resolve the taxonomic status of some species (Mack and Kvist 2019, Kaygorodova et al. 2020). For example, the molecular studies, conducted by Siddall et al. (2005) and Mack and Kvist (2019) on North American populations previously assigned to *Glossiphoniacomplanata* (Linnaeus, 1758), reveal the presence of two well-defined species, with *Glossiphoniacomplanata* restricted to Europe and *Glossiphoniaelegans* (Verrill, 1872) living in North America. However, the knowledge on diversity and species delimitation by applying a molecular genetic approach within this leech group is still poorly studied in many parts of their range, especially in the Dinaric Region of the Balkan Peninsula.

At present, all of the European members of the genus *Glossiphonia* have been reported to also inhabit the Western Balkans (Sket 1968, Nesemann and Neubert 1999, Utevsky et al. 2013, Grosser et al. 2015a, Grosser et al. 2015b, Dmitrović and Pešić 2020): the widespread Palearctic *G.complanata*, with two subspecies, the nominal one and *G.complanatamaculosa* Sket, 1968 which is known only from Ohrid Lake, *G.concolor* (Apathy, 1988), *G.nebulosa* Kalbe, 1964, *G.paludosa* (Carena, 1824), *G.balcanica* Grosser & Pešić, 2016, a species recently described from Kosovo and Montenegro (Grosser et al. 2016) and *G.pulchella* Sket, 1968 an endemic species known only from the littoral of Lake Ohrid (Sket 1968).

In this study, we applied a standard DNA barcoding marker, a fragment of the COI gene, to analyse specimens of the genus *Glossiphonia* collected recently in various freshwater habitats (lakes, streams and springs) of the Western Balkans (Albania, Bosnia and Hercegovina, Kosovo, Montenegro and North Macedonia). In addition, we analysed the available museum material originating from other European localities, including loci typici of some selected species (e.g. *G.nebulosa*) to obtain reliably identified sequence data. Moreover, we used DNA barcode sequences in both BOLD and GenBank to compare with the sequences obtained in our study. As a result, a dataset, including COI sequences of 29 specimens of *Glossiphonia* spp. plus four sequences representing two other genera (*Helobdella*, *Placobdella* Blanchard, 1893), was generated in order to contribute to a reference dataset applicable for DNA barcoding studies of the genus *Glossiphonia* in general and, in particular, in the Western Balkans.

## Material and Methods

### Sample collection and morphological analysis

**Figure 1.**
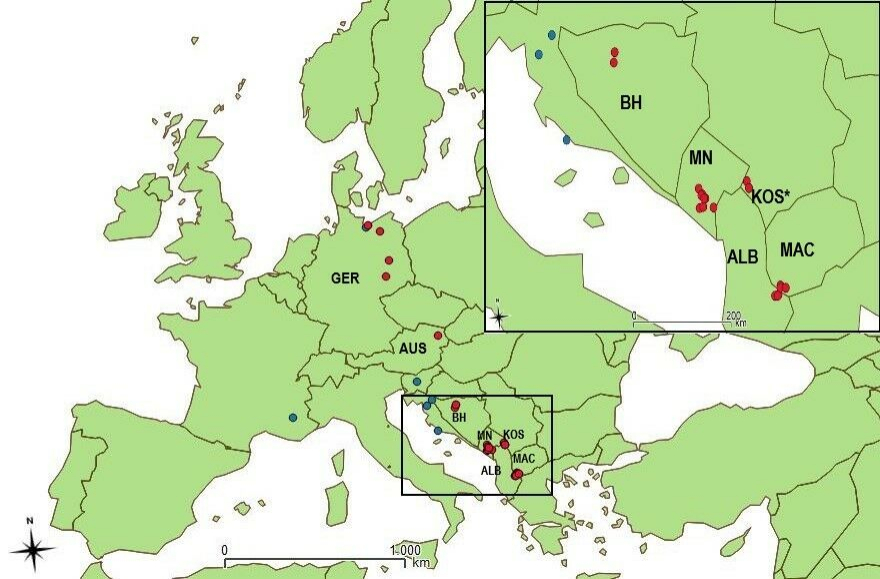
Distribution map of localities where glossiphoniid leeches were collected. (red dots – present study records, blue dots – records from previous studies where coordinates are available). Note that each point may represent more than one species. Country codes of those countries, from which we had material, are indicated. The map was created using QGIS 2.8.11 software.

Glossiphoniid leeches were collected from twenty-two sites in seven countries: Albania, Austria, Bosnia and Hercegovina, Germany, Kosovo, Montenegro and North Macedonia (Fig. 1). Leeches were collected by tweezers from the underside of hard substratum (stones, wood) and on plants submerged in the water, on banks, as well as on the shore. Material was preserved in 96% ethanol for further morphological and molecular genetic analysis.

Morphological analysis of 33 individuals was performed using a stereomicroscope (Novex). Leeches were identified to species level according to Nesemann and Neubert (1999) and Grosser et al. (2016). Voucher specimens were deposited in the scientific collection of the Natural History Museum Vienna (NHM).

### Molecular genetic analysis

DNA analysis was conducted in the Central Research Laboratories of the NHM. Leeches fixed in 96% ethanol were stored at 4°C. Tissue samples from individuals (approx. 2 × 2 × 2 mm) were separated using sterile scalpels and tweezers. DNA was extracted with the DNeasy Blood and Tissue Kit (Qiagen, Hilden, Germany) according to the manufacturer's protocol. The final volume of DNA solution was 40 µl.

The present study focused on the COI gene, which was amplified using a polymerase chain reaction (PCR). For all sampled leeches, a 708 bp section was amplified, which contains the standard DNA barcoding region. The universal primers LCO1490, 5′GGTCAACAAATCATAAAGATATTGG-3′ and HCO2198, 5′TAAACTTCAGGGTGACCAAAAAATCA-3′ (Folmer et al. 1994) were used. The final alignment for the phylogenetic tree reconstructions included 33 sequences and had a length of 658 sites.

Each reaction consisted of 0.5 units of TopTaq DNA polymerase (Qiagen), 2.5 µl 10× TopTaq PCR Buffer, 10 mM of each dNTP, 50 µM of each primer and 1 µl DNA template in a total reaction volume of 25 µl. The PCR cycling protocol included an initial denaturation at 94°C for 3 min, followed by 35 cycles of of denaturation at 94°C for 30 s, annealing for 30 s at 52°C and extension for 1 min at 72°C. The final step was an extension at 72°C for 10 min and a hold at 10°C.

The amplicons were checked by (1%) agarose gel electrophoresis. The QIAquick PCR Purification Kit (Qiagen) was employed to purify amplifications products. Sequencing was performed in both directions at Microsynth (Balgach, Switzerland) using the PCR primers.

### Data analysis

Sequences (both strands) were checked and edited using BioEdit (Hall 1999). The search in GenBank for sequences similar to the sequences, generated from the studied specimens analysed in the present study, was performed through BLASTn search in the GenBank database (https://blast.ncbi.nlm.nih.gov). Subsequently, 19 published sequences, from representatives of the genus *Glossiphonia*, were downloaded from GenBank (https://www.ncbi.nlm.nih.gov/genbank) and were included for comparison (listed in Table 1). Differences between DNA sequences (p and K2P distances in %) were calculated with the MEGA X software, version 10.1.7 (Kumar et al. 2018). MEGA X was also used to calculate a Neighbour-Joining (NJ,Saitou and Nei 1987) tree (based on p distances) and Maximum Likelihood (ML) trees (model selected by the BIC criterion (Bayesian Information Criterion) implemented in MEGA X: TN93 + I+ G) with an initial NJ tree and using the Subtree-Pruning-Regrafting - Extensive heuristic search (SPR level 5). Bootstrapping was done with 500 replicates for NJ and ML trees. A Bayesian Inference (BI) tree was calculated with MrBayes v.3.2.2 (Huelsenbeck and Ronquist 2001, Ronquist et al. 2012) with 10^6^ generations (two runs each with four chains and one heated chain, sampling every hundredth tree). The first 25% of the trees were discarded as burn-in and a 50% majority rule consensus tree was calculated from the remaining trees.

In order to assess the genetic differentiation of species within our dataset of 47 *Glossiphonia* sequences, we used the ASAP procedure designated to a list of partitions of species hypotheses using genetic distances, calculated between DNA sequences and ranked by their ASAP-scores: the lower the score, the better the partition (Puillandre et al. 2021). The online ASAP version (https://bioinfo.mnhn.fr/abi/public/asap/asapweb.html) was used, with default settings and the K2P distance model. Besides the ASAP procedure, we also used the species delimitation approach of mPTP by Kapli et al. (2017), which is based on a single-locus coalescent-based method. All sequences, generated in course of the present study, were deposited in BOLD.

## Results

### Morphological characterisation of collected specimens

**Figure 2.**
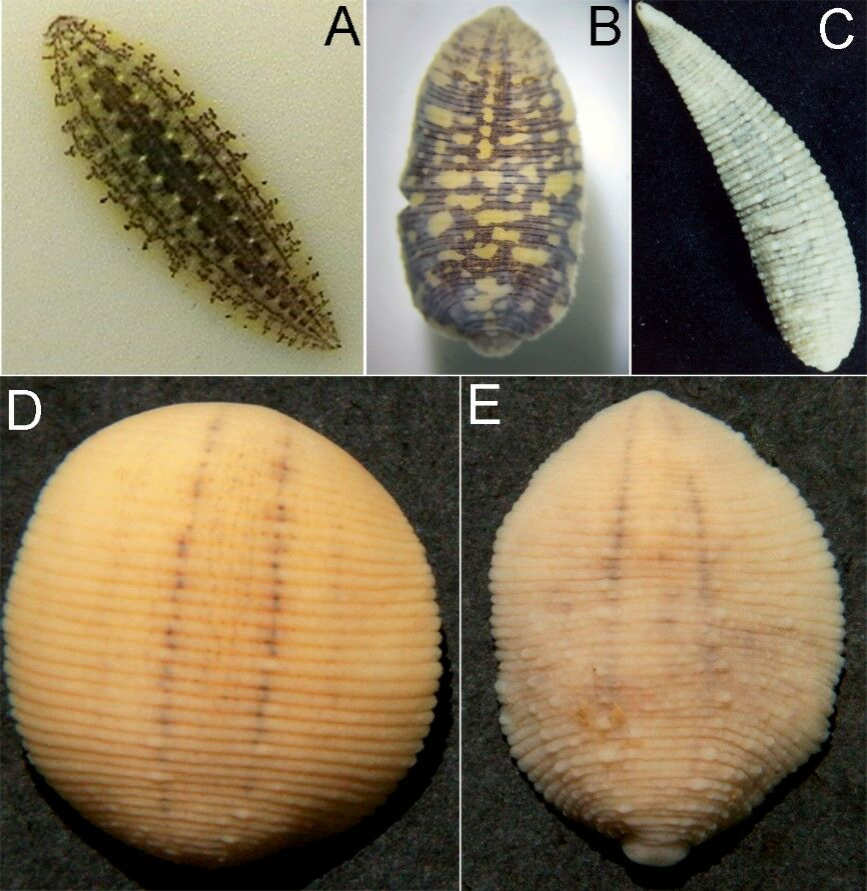
Photographs of selected leeches of *Glossiphonia*. **A** *G.c.complanata*, river Helme near Bennungen, Germany; **B** *G.c.maculosa*, Ohrid Lake, North Macedonia; **C** *G.nebulosa*, River Helme near Bennungen, Germany; **D** *G.balcanica*, Toplla spring near Dečani, Kosovo; **E** G.cf.nebulosa, Toplla spring near Dečani, Kosovo. Photos: C. Grosser (A, D-E), V. Pešić (B), J. Händel (C).

Morphological analysis of 29 specimens of the leech genus *Glossiphonia* from studied area of West Balkans revealed the presence of three species *G.complanata*, *G.balcanica* and *G.nebulosa* (Fig. 2), which could be differentiated morphologically by the following characters: the six-eyed leeches with prominent papillae only on annulus a2 of mid-body somites were assigned to the typical *G.complanatacomplanata*. Specimens from Lake Ohrid and Lake Prespa were identified as *G.complanatamaculosa*. Representatives of this subspecies can be separated from the nominal subspecies by the lack of the prominent dorsal papillae, characteristic for *G.complanatacomplanata* and by the presence of a brown pigmentation forming an asymmetrical reticulate pattern, often covering completely the body surface (Nesemann and Neubert 1999). Although *G.c.maculosa* was so far known from Lake Ohrid only, the morphological determination of the specimens from Lake Prespa was straightforward.

*Glossiphoniabalcanica* and *G.nebulosa* closely resemble one another and can be distinguished by the colour, which is bright brownish in *G.balcanica*, but more greyish in the specimens of *G.nebulosa* from the Balkans (see Grosser et al. 2016). Moreover, the dorsal surface of *G.balcanica* is covered by a few small irregularly arranged papillae and the prominent paramedian papillae located only on annulus a2. Finally, a difference between those two species can be found in the medial fold on the cranial sucker. This medial fold is lacking in *G.balcanica* specimens, but is prominent in the population of G.cf.nebulosa from Kosovo and only slightly developed in the populations of *G.nebulosa* from its type locality in Germany (see Grosser et al. 2016 for further discussion).

### Molecular genetic analysis

The final alignment comprised 52 sequences and had a length of 658 nucleotide sites. Of the 33 COI sequences generated in the present study, all were included in the final dataset for the phylogenetic analysis.

**Figure 3.**
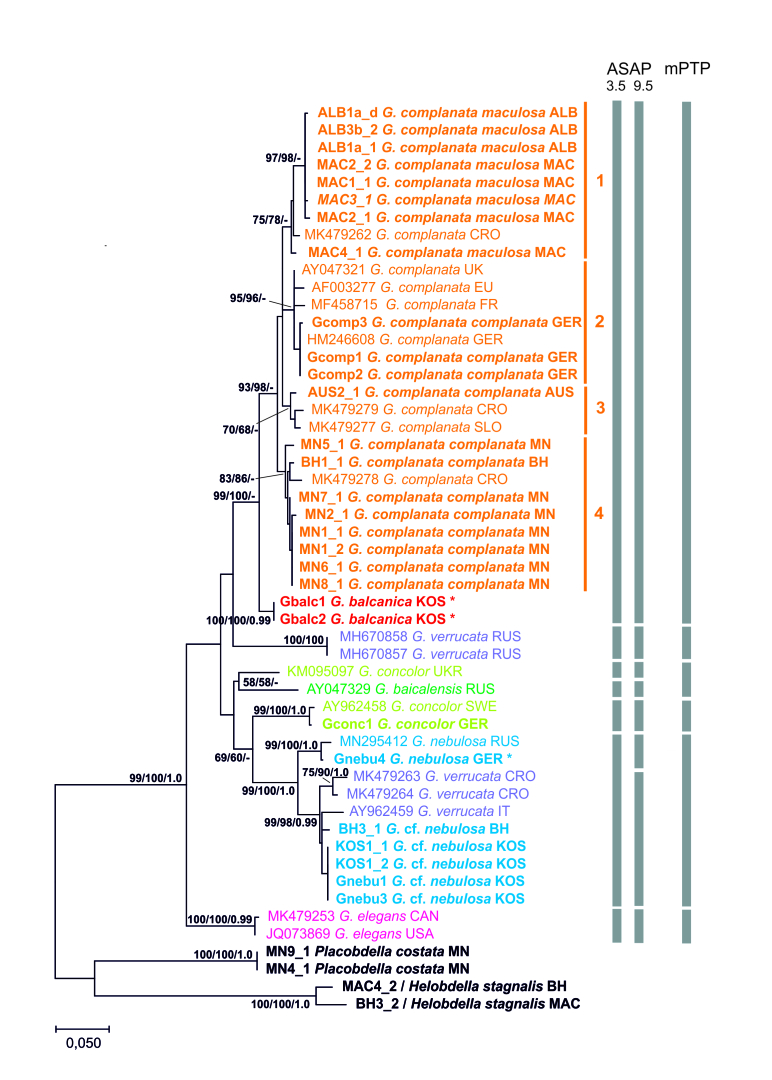
Maximum Likelihood tree of Glossiphoniidae, obtained from 52 nucleotide COI sequences. Bootstrap values > 50% are provided at major nodes for both tree calculation methods (ML/NJ). In addition, posterior probability values ≥ 0.98 of the BI analysis are provided (third value). The results of species delimitation are indicated by vertical bars. Sequences generated in the course of the present study are given in bold. Country codes are the same as in Table 1. Asterisks mark specimens from the type locality.

Both the ML and NJ trees, based on COI sequences, were in agreement regarding the general topology. The ML tree is shown in Fig. 3 (NJ bootstrap values, as well as posterior probability values of the BI analysis are also shown in Fig. 3). Although the BI tree was, in general, very weakly supported and had a slightly different topology (see below), several clades were consistent with the ML and NJ trees. The species of the genus *Glossiphonia* form a monophylum. *Placobdela* and *Helobdella*, which were included as outgroup, form separate sister clades (bootstrap support > 96%).

In both the NJ and ML trees, *Glossiphonia* COI sequences are clustered into eight clades (Fig. 3). The North American *G.elegans* forms the sister group to a clade comprising all European members of the genus analysed. Yet, the latter group is supported by low bootstrap support only (ML: 49%). In the BI analysis, this sister group relationship was not found and, in general, the relationships amongst clades were only weakly supported in the BI tree. In the following, the ML tree (Fig. 3) regarding European *Glossiphonia* is described. A large clade represents *Glossiphoniacomplanata*, which is subdivided into four subclades. *Glossiphoniac.maculosa* specimens collected from Ohrid Lake (from Albania and North Macedonia) are clustered together (supported by an ML bootstrap value of 97%) and are most closely related to two other *G.complanata* specimens, one *Glossiphoniac.complanata* (MK479262), collected from Gacka River, Otočac, Croatia and the other one, a specimen of *G.complanatamaculosa* from Prespa Lake, North Macedonia (LCHME024-20). Thus, with the exception of the Croatian specimen, *G.c.maculosa* would be an (albeit very weakly supported) monophylum (Subclade 1 in Fig. 3). The range of K2P distances between *G.c.maculosa* from the Balkan lakes and *G.c.complanata* (excluding the individual MK479262 from Croatia) was 2.07 ± 0.5%. The remaining (weakly to moderately supported) *G.complanata* subclades cluster to some extent in a geographic manner: the (predominately more western) Subclade 2 contains seven individuals collected from Germany, France and the United Kingdom. Subclade 3 comprises individuals from Austria, Croatia and Slovenia and Subclade 4 contains individuals from Bosnia & Herzegovina, Croatia and Montenegro. Despite bad support values, the BI tree also revealed Subclades 1, 2 and 4.

*Glossiphoniabalcanica*, in our tree represented by two specimens from Kosovo, is the sister clade of *G.complanata*. This sister group relationship was supported with high support values. The third species in our dataset is *G.concolor*, of which we had only one sample from Mecklenburg-Vorpommern, Germany (LCHME041-20). This sequence is very similar to the GenBank sequence of *G.concolor* from Sweden. In contrast, another published sequence (KM095097) from Ukraine is quite distantly related, clustering in the tree (albeit with no considerable support) with a published sequence of *G.baicalensis* (from Lake Baikal, Russia; AY047329). Another distinct lineage is formed by two GenBank sequences of *Glossiphoniaverrucata* from Russia.

For the fourth species sequenced in the present study, *G.nebulosa*, we found two subclades, one formed by a specimen from the type locality in Germany (LCHME044-20), together with a specimen from Russia (MN295412). This clade is placed with high support (99%) as the sister group of a clade consisting of *G.nebulosa* specimens from Bosnia and Herzegovina, as well as Kosovo (in the tree designated as G.cf.nebulosa). Moreover, this subclade contains published sequences assigned to *G.verrucata*, originating from Croatia (MK479263-64) and Italy (AY96245) and rendering *G.verrucata* paraphyletic. Due to the position of *G.verrucata* specimens within the *G.nebulosa* clade, this species also appears paraphyletic.

Species from the two genera, *Placobdela* and *Helobdella*, analysed in this study as outgroup species, formed separate clades with a bootstrap support of 99%. Two specimens of *P.costata* are identical (BOLD BIN: AEC5178), while the two specimens determined as *H.stagnalis* are separated by a *p*-distance of 4.1% and also have separate BINs in BOLD.

### Genetic distances and delimitation of species

The mean K2P values between the morphologically determined species of *Glossiphonia* ranged from 3.17% to 12.69% (Suppl. material 1). The minimal mean K2P distance of 3.17 ± 0.6% was found between *G.balcanica* from Kosovo and the *G.complanata* clade. The maximum mean distance of 12.69 ± 1.6% was observed between G.cf.nebulosa clade and the Siberian *G.verrucata*. A mean distance of 5.07 ± 0.8% separates *G.nebulosa* (comprising one sequence from Germany and one from Russia) from a clade consisting of specimens from the Balkan Region (including specimens here provisionally assigned to G.cf.nebulosa).

The highest mean intraspecific distances were observed within *G.complanata* (1.64%, max. 3.0%) and G.cf.nebulosa (1.31%, max. 4.0%), respectively. The mean intraspecific distance within the clade, herein labelled as *G.nebulosa*, amounted to 0.9% (Suppl. material 1).

For the ASAP analysis, the sequences of *G.verrucata* from Italy (AY962459) were excluded from the further analysis because of having ambiguous nucleotides (see Kaygorodova et al. (2020) for a discussion). As a result of the ASAP analysis, a barcoding gap at about 4-8% was estimated. The applied ASAP procedure identified 7 MOTUs (hypothetical species) at the threshold distance of 5.46% (K2P) which has the best ASAP‐score (3.50) within the available molecular data: *G.complanata* (merging *G.balcanica*), *G.concolor*, *G.concolor* from Ukraine, *G.baicalensis*, *G.elegans*, *G.nebulosa* and *G.verrucata*. At the threshold distance of 3.59% (K2P) (but with a poorer ASAP‐score of 9.50), ASAP analysis retrieved one more *Glossiphonia* species (hereafter referred to as G.cf.nebulosa) from the Balkans, morphologically resembling *G.nebulosa*.

Finally, the mPTP analysis grouped the *Glossiphonia* COI sequences into six main species also combining *G.complanata* and *G.balcanica*. In contrast to the ASAP results, the highly diverged lineages of *G.baicalensis* and *G.concolor* were grouped into one species (Fig. 3).

## Discussion

Morphological analysis of the examined leeches of the genus *Glossiphonia* from the Western Balkans revealed the presence of three species, *G.complanata*, *G.balcanica* and *G.nebulosa*. Using DNA barcodes, the present study has revealed inconsistency between the past understanding of the taxonomic diversity of the above-listed three species, based exclusively on morphological characters.

*Glossiphoniacomplanata* was the most abundant species in our study. In the studied area, it is known by two subspecies, the nominal one and *G.complanatamaculosa*, known only from Lake Ohrid (Sket 1968). Surprisingly, the results of our study revealed that a published sequence of a specimen of *G.complanata* (MK479262) from Croatia is close to the *G.complanatamaculosa* subclade from Ohrid Lake. The latter specimen, reported by Mack and Kvist (2019), was collected from the Gacka River in Otočac, Croatia. The authors emphasised the high genetic variation found between the specimen from Otočac and other specimens of *G.complanata* from Croatia, suggesting that Otočac’s population represent a separately evolving lineage (Mack and Kvist 2019). Future morphological analysis of those specimens should allow us to test if morphology supports the genetic results.

In the course of the present study, only a single *Glossiphonia* specimen was found in Prespa Lake (MAC4_1; LCHME024-20) which was assigned to *G.complanatamaculosa*, based on its characteristic colour pattern. Albrecht et al. (2008) emphasised a high degree of isolation of Lake Ohrid endemics with relatively little faunal overlap with the neighbouring Prespa Lake. The COI analysis revealed that the specimen from Lake Prespa clusters with the samples from Ohrid Lake, but is separated by 1.49% mean K2P distance from that *maculosa*-lineage, implying some level of genetic isolation of the populations from these two Balkan Lakes. Yet, more samples, including Croatian localities, should be needed to support this assumption and to assess morphological variation within this clade.

Our results suggest that some specimens, represented by published sequences in our dataset, were probably misidentified. Sequences of two samples from one unnamed river in Croatia (Mack and Kvist 2019) and a sequence from another sample from Rio Sadde, Italy (Siddall et al. 2005), all reported under the name *G.verrucata*, were found within the G.cf.nebulosa clade in our study. Already Kaygorodova et al. (2020) showed that the latter sample from Italy is genetically clearly separated from their Siberian leeches that morphologically match *G.verrucata*, a species recently re-described by Jueg and Michalik (2018). The latter species is distributed mainly in the northern Palaearctic (Jueg 2013) with a few records from Central Europe where it was found in the River Danube from Bavaria to Hungary (Nesemann 1997, Nesemann and Neubert 1999). The results of sequence-based species delimitation methods, conducted by Kaygorodova et al. (2020), revealed that specimens from Italy and Russia (Siberia), respectively, represent distinct species. Unfortunately, for the samples from Italy and Croatia, published under the name of *G.verrucata* by Siddall et al. (2005) and Mack and Kvist (2019), respectively, no information on their morphological features used for their identification were provided. The taxonomic state of this clade (in the present study, summarised under G.cf.nebulosa) deserve further investigation.

Our study revealed that *G.nebulosa* consists of two phylogenetic clades questioning the status of the populations from Western Balkans. The mean K2P distance of about 4.9% was found between the north-central European clade of *G.nebulosa* containing a specimen from the type locality of this species in Germany (stream Nieplitz near Berlin) and the clade that encompasses specimens from the Balkans. This might suggest longstanding isolation between populations from north-central Europe and populations from south-eastern Europe. Grosser et al. (2016) had stressed the morphological differences between the populations of *G.nebulosa* from its type locality*l*and those from Kosovo. To clarify the taxonomical status of the latter populations from the West Balkans, further material should be sampled and studied to cover the distribution ranges of these taxa.

Concerning the species delimitation analyses, the different results of the approaches did not provide convincing conclusions. For example, the mPTP analysis combined *G.complanata* and *G.balcanica* into one species which is rather unlikely, comparing the distance between these two lineages (6.14% K2P) with other inter- and intraspecific distances in the genus. Another unexpected result relates to *G.complanata* and *G.balcanica*: mean K2P interspecific distance between *G.balcanica*, an endemic species recently described by Grosser et al. (2016) from Kosovo and the widely distributed *G.complanata*, amounted to 3.17%. The ASAP procedure, grouped the COI sequences of the latter two species together, which is not consistent with the morphological differences between these two species. Morphologically, *G.balcanica* is rather different from *G.complanata* and closely resembles G.cf.nebulosa, with which it lives syntopically at some localities (see Grosser et al. 2016). Phylogenetically, *G.balcanica* and *G.complanata* belong to different clades with high support values indicating that the interspecific threshold in ASAP analysis of our analysed dataset might be unrealistic, likely as a result of an underestimation of species diversity (Yu et al. 2017) or estimating a relatively large barcoding gap in species which diverged recently (Kvist et al. 2010). According to Puillandre et al. (2021) ASAP is most effective when species are represented by at least 3–5 sequences. It could be that only having two sequences of *G.balcanica* vs. the multitude of sequences for *G.complanata* could have resulted in a merging of the two species. In summary, as recently emphasised by Puillandre et al. (2021), other characters and not just threshold distances with the best score, should be used to select a final species partition, in the sense of integrative taxonomy.

The obtained barcode gaps of 4–8% K2P in our dataset of COI sequences is comparable to an interspecific threshold of 5–7% distance that Kaygorodova et al. (2020) accepted as suitable for species identification purposes of Siberian glossiphonid leeches. The results of our study emphasised the importance of molecular genetic methods and of assessing genetic diversity of glossiphoniid leeches. Building of the DNA barcode reference library for this group will provide a handy system for better understanding of distribution and species boundaries of this genus in karstic regions of the Western Balkans.

## Supplementary Material

2A0A4386-8BEF-505A-8E37-6A19589EA67D 10.3897/BDJ.9.e66347.suppl1 Supplementary material 1 Table S1. Data type Table Brief description Interspecific mean K2P distances (below diagonal) and mean *p*-distances (above diagonal) and the standard deviations. Diagonal: the ranges of intraspecific genetic divergence are marked in bold font (the mean values are given in parentheses; K2P and *p*-distance gave similar results). The number of specimens considered for each species is indicated in parentheses. In the group of G.cf.nebulosa (n = 8), the three *G.verrucata* sequences from Italy and Croatia were included for the calculations. File: oo_543534.docx https://binary.pensoft.net/file/543534 Milica Jovanović, Elisabeth Haring, Helmut Sattmann, Vladimir Pešić

## Figures and Tables

**Table 1.** Taxon names, locality information and accession numbers for the specimens used in phylogenetic analysis and distance estimations. Newly-sequenced taxa are shown in bold font. BOLD accession numbers are given for the sequences produced in the present study, while GenBank accession numbers are provided for published sequences.

**Sample ID**	**Locality (Country/Exact site)**	**Coordinates**	**BOLD** / **GenBank ID**	**Source**
*** Glossiphonia verrucata ***				
ROMIZ I11753	Unnamed river, Croatia (CRO)	43.574722°N, 15.818889°E	MK479263	Mack and Kvist (2019)
ROMIZ I11755	Unnamed river, Croatia (CRO)	43.574722°N, 15.818889°E	MK479264	Mack and Kvist (2019)
	Rio Sadde, Italy (IT)		AY962459	Siddall et al. (2005)
	Chechuy River, Russia (RUS)	58.194640°N, 109.294720°W	MH670857	Kaygorodova et al. (2020)
	Lake near Meget, Russia (RUS)	52.451440°N, 104.027120°W	MH670858	Kaygorodova et al. (2020)
*** Glossiphonia complanata complanata ***				
BH1_1	Krupa River near Vrbas, Bosnia and Hercegowina (BH)	44.616°N, 17.1495°E	**LCHME001-20**	This study
MN1_1	Karuč spring, Podgorica, Montenegro (MN)	42.3585°N, 19.1064°E	**LCHME008-20**	This study
MN1_2	Karuč spring, Podgorica, Montenegro (MN)	42.3585°N, 19.1064°E	**LCHME009-20**	This study
MN2_1	River Crnojevića, Cetinje, Montenegro (MN)	42.3546°N, 19.0178°E	**LCHME010-20**	This study
MN5_1	Vitoja spring pool, Podgorica, Montenegro (MN)	42.3251°N, 19.3634°E	**LCHME013-20**	This study
MN6_1	Dobro polje spring, Danilovgrad, Montenegro (MN)	42.6305°N, 19.0324°E	**LCHME014-20**	This study
MN7_1	Mareza spring, Podgorica, Montenegro (MN)	42.48°N, 19.1822°E	**LCHME015-20**	This study
MN8_1	Karuč spring, Podgorica, Montenegro (MN)	42.3585°N, 19.1064°E	**LCHME016-20**	This study
AUS_Hir:2_1	Kalte Wien, Vienna, Austria (AUS)	48.2934°N, 16.3915°E	**LCHME027-20**	This study
Gcomp1	Stream from the lake Barschsee, Mecklenburg-Vorpommern, Germany, type locality (GER)	53.9147°N, 11.2815°E	**LCHME038-20**	This study
Gcomp2	Stream from the lake Barschsee, Mecklenburg-Vorpommern, Germany, type locality(GER)	53.9147°N, 11.2815°E	**LCHME039-20**	This study
Gcomp3	Small stream near Jesewitz, Saxony, Germany (GER)	51.3812°N, 12.6733°E	**LCHME040-20**	This study
	Durance river, France (FR)		MF458715	Corse et al. (2017)
	Europe (EU)		AF003277	Siddall and Burreson (1998)
	Creek, Mecklenburg-Vorpommern, Nordwestmecklenburg district, Germany (GER)	53.81848°N, 10.92799°E	HM246608	Trajanovski et al. (2010)
	United Kingdom (UK)		AY047321	Light and Siddall (1999)
ROMIZ I11750	Korana river, Croatia (CRO)	45.117222°N, 15.592778°E	MK479280	Mack and Kvist (2019)
ROMIZ I11749	Korana river, Croatia (CRO)	45.117222°N, 15.592778°E	MK479279	Mack and Kvist (2019)
ROMIZ I11748	Korana river, Croatia (CRO)	45.117222°N, 15.592778°E	MK479278	Mack and Kvist (2019)
ROMIZ I11717	Sava river, Slovenia (SLO)	46.084444°N, 14.587222°E	MK479277	Mack and Kvist (2019)
ROMIZ I11743	Gacka river, Croatia (CRO)	44.851667°N, 15.233611°E	MK479262	Mack and Kvist (2019)
*** Glossiphonia complanata maculosa ***				
MAC1_1	St. Naum spring of Crni Drim, Ohrid Lake, North Macedonia (MAC) – type locality	40.9138°N, 20.7433°E	**LCHME020-20**	This study
MAC2_1	Lagadin, Ohrid Lake, North Macedonia (MAC)	41.0422°N, 20.8039°E	**LCHME021-20**	This study
MAC2_2	Lagadin, Ohrid Lake, North Macedonia (MAC)	41.0422°N, 20.8039°E	**LCHME022-20**	This study
MAC3_1	Peštani, Ohrid Lake, North Macedonia (MAC)	41.0095°N, 20.8059°E	**LCHME023-20**	This study
MAC4_1	Oteševo, Prespa Lake, North Macedonia (MAC)	40.9919°N, 20.9322°E	**LCHME024-20**	This study
ALB1a_d	Pogradec, Ohrid Lake, Albania (ALB)	40.9058°N, 20.6556°E	**LCHME029-20**	This study
ALB1a_1	Pogradec, Ohrid Lake, Albania (ALB)	40.9058°N, 20.6556°E	**LCHME030-20**	This study
ALB3b_2	Tushemisht, Ohrid Lake, Albania (ALB)	40.9035°N, 20.7172°E	**LCHME035-20**	This study
*** Glossiphonia concolor ***				
Gconc1	Krakower Obersee, Mecklenburg-Vorpommern, Germany (GER)	53.6074°N, 12.2976°E	**LCHME041-20**	This study
	Kila river, Sweden (SWE)		AY962458	Siddall et al. (2005)
	Ukraine (UKR)		KM095097	Kaygorodova and Mandzyak (2014)
*** Glossiphonia balcanica ***				
Gbalc1	Toplla spring, Dečani, Kosovo (KOS) - type locality	42.57194°N, 20.29056°E	**LCHME036-20**	This study
Gbalc2	Toplla spring, Dečani, Kosovo (KOS) - type locality	42.57194°N, 20.29056°E	**LCHME037-20**	This study
*** Glossiphonia nebulosa ***				
Gnebu4	Berliner Chausssee stream Nieplitz, Berlin, Germany -type locality	52.1348°N, 12.9449°E	**LCHME044-20**	This study
	Yamalo-Nenets Autonomous Okrug, Russia (RUS)		MN295412	Bolotov et al. (2019)
KOS1_1	Spring KS 40, Peje, Kosovo (KOS)	42.6283°N, 20.246°E	**LCHME004-20**	This study
KOS1_2	Spring KS 40, Peje, Kosovo (KOS)	42.6283°N, 20.246°E	**LCHME005-20**	This study
BH3_1	Banja Luka, Near castle, Bosnia & Herzegovina (BH)	44.7657°N, 17.193°E	**LCHME002-20**	This study
Gnebu1	Toplla spring, Dečani, Kosovo (KOS)	42.57194°N, 20.29056°E	**LCHME042-20**	This study
Gnebu3	Toplla spring, Dečani, Kosovo (KOS)	42.57194°N, 20.29056°E	**LCHME043-20**	This study
*** Glossiphonia elegans ***				
ROMIZ I11505	Unknown pond, Nopiming, Manitoba, Canada (CAN)	50.452222°N, 95.5125°W	MK479253	Mack and Kvist (2019)
	Lake Bemidji, Beltrami County, Minnesota, (USA)		JQ073869	Moser et al. (2012)
*** Glossiphonia baicalensis ***				
	Lake Baikal, Russia (RUS)		AY047329	Light and Siddall (1999)
*** Outgroups ***				
*** Placobdella costata ***				
MN4_1	Oraška jama spring, Danilovgrad, Montenegro (MN)	42.5309°N, 19.0921°E	**LCHME012-20**	This study
MN9_1	Crno oko spring, Podgorica, Montenegro (MN)	42.4844°N, 19.1542°E	**LCHME017-20**	This study
*** Helobdella stagnalis ***				
BH3_2	Near castle, Banja Luka, Bosnia & Herzegovina (BH)	44.7657°N, 17.193°E	**LCHME003-20**	This study
MAC4_2	Oteševo, Prespa Lake, North Macedonia (MAC)	40.9919°N, 20.9322°E	**LCHME025-20**	This study
