# Supplementary material for: DNA barcoding for species delimitation of the freshwater leech genus Glossiphonia from the Western Balkan (Hirudinea, Glossiphoniidae)
Source: Biodivers Data J. 2021 Sep 15;9:e66347. doi: 10.3897/BDJ.9.e66347 (PMC8458266; doi:10.3897/BDJ.9.e66347)
Supplement: Supplementary material 1 — Table S1. [file bdj-09-e66347-s001.docx]

|  | | **Clades** | | | | | | | | | | | |
| --- | --- | --- | --- | --- | --- | --- | --- | --- | --- | --- | --- | --- | --- |
|  |  | 1 | 2 | 3 | 4 | 5 | 6 | 7 | 8 | 9 | 10 | 11 |  |
| 1 | *G. complanata* (n=28) | **0-3.0 (1.64)** | 3.08±0.61 | 8.29±1.1 | 7.29±1.0 | 7.20±1.0 | 9.42±1.2 | 9.75±1.2 | 9.93±1.2 | 10.29±1.2 | 19.31±1.6 | 16.03±1.5 |  |
| 2 | *G. balcanica* (n=2) | 3.17±0.6 | **0 (0)** | 8.35±1.2 | 5.88±1.0 | 6.26±1.0 | 9.30±1.2 | 9.49±1.2 | 9.84±1.2 | 9.68±1.2 | 18.79±1.6 | 15.56±1.5 |  |
| 3 | *G. verrucata* RUS (n=2) | 8.80±1.3 | 8.86±1.3 | **0 (0)** | 9.30±1.2 | 10.25±1.3 | 10.82±1.3 | 11.01±1.3 | 11.62±1.3 | 11.20±1.2 | 21.35±1.7 | 17.46±1.6 |  |
| 4 | *G. concolor* UKR (n=1) | 7.68±1.1 | 6.13±1.1 | 9.94±1.4 | **(n/c)** | 5.88±1.0 | 8.35±1.2 | 8.92±1.2 | 9.30±1.3 | 9.96±1.3 | 20.30±1.7 | 17.08±1.6 |  |
| 5 | *G. baicalensis* (n=1) | 7.59±1.2 | 6.55±1.2 | 11.05±1.5 | 6.14±1.1 | **(n/c)** | 9.01±1.2 | 8.54±1.2 | 9.23±1.3 | 10.15±1.3 | 19.83±1.7 | 16.13±1.5 |  |
| 6 | *G. concolor* GER, SWE (n=2) | 10.12±1.4 | 9.98±1.4 | 11.73±1.6 | 8.94±1.4 | 9.68±1.4 | **0-0.3** (**0.3)** | 9.68±1.2 | 8.61±1.3 | 11.29±1.3 | 21.63±1.7 | 17.46±1.6 |  |
| 7 | *G. nebulosa* (n=2) | 10.47±1.4 | 10.17±1.4 | 11.95±1.6 | 9.54±1.4 | 9.14±1.4 | 10.43±1.4 | **0-0.9** (**0.9)** | 4.89±1.3 | 11.10±1.3 | 22.49±1.8 | 17.17±1.5 |  |
| 8 | *G*. cf. *nebulosa* (n=8) | 10.72±1.4 | 10.63±1.4 | 12.69±1.6 | 10.04±1.4 | 9.92±1.4 | 9.19±1.3 | 5.07±0.8 | **0-4.0** (**1.31)** | 10.82±1.3 | 22.30±1.8 | 17.10±1.6 |  |
| 9 | *G. elegans* (n=2) | 11.11±1.5 | 10.40±1.5 | 12.14±1.6 | 10.76±1.5 | 10.95±1.5 | 12.29±1.7 | 12.05±1.6 | 11.76±1.5 | **0-0.3** (**0.19)** | 20.49±1.7 | 16.98±1.5 |  |
| 10 | *Helobdella stagnalis* (n=2) | 18.07±2.1 | 17.46±2.2 | 19.90±2.4 | 19.42±2.4 | 18.16±2.3 | 19.90±2.5 | 19.53±2.6 | 19.47±2.5 | 19.32±2.4 | **0-4.1** (**4.1)** | 20.21±1.7 |  |
| 11 | *Placobdella costata* (n=2) | 22.41±2.0 | 21.67±2.0 | 25.28±2.1 | 23.72±2.1 | 23.05±2.1 | 25.63±2.2 | 26.84±2.1 | 26.58±2.1 | 24.02±2.1 | 23.63±2.4 | **0 (0)** |  |
